# Restoring synaptic plasticity and memory in mouse models of Alzheimer’s disease by PKR inhibition

**DOI:** 10.1186/s13041-017-0338-3

**Published:** 2017-12-13

**Authors:** Kyoung-Doo Hwang, Myeong Seong Bak, Sang Jeong Kim, Sangmyung Rhee, Yong-Seok Lee

**Affiliations:** 1 Department of Life Science, College of Natural Science, Chung-Ang University, Seoul, 06974 Republic of Korea; 2 Department of Physiology, Seoul National University College of Medicine, Seoul, 03080 Republic of Korea; 3 Department of Biomedical Sciences, Seoul National University College of Medicine, Seoul, 03080 Republic of Korea; 4 Neuroscience Research Institute, Seoul National University College of Medicine, Seoul, 03080 Republic of Korea

**Keywords:** Alzheimer’s disease (AD), Amyloid β (Aβ), PKR inhibitor (PKRi), Contextual fear conditioning, Object recognition memory, Long-term potentiation (LTP)

## Abstract

Alzheimer’s disease (AD) is a neurodegenerative disorder associated with deficits in cognition and synaptic plasticity. While accumulation of amyloid β (Aβ) and hyper-phosphorylation of tau are parts of the etiology, AD can be caused by a large number of different genetic mutations and other unknown factors. Considering such a heterogeneous nature of AD, it would be desirable to develop treatment strategies that can improve memory irrespective of the individual causes. Reducing the phosphorylation of eukaryotic translation initiation factor 2α (eIF2α) was shown to enhance long-term memory and synaptic plasticity in naïve mice. Moreover, hyper-phosphorylation of eIF2α is observed in the brains of postmortem AD patients. Therefore, regulating eIF2α phosphorylation can be a plausible candidate for restoring memory in AD by targeting memory-enhancing mechanism. In this study, we examined whether PKR inhibition can rescue synaptic and learning deficits in two different AD mouse models; 5XFAD transgenic and Aβ_1–42_-injected mice. We found that the acute treatment of PKR inhibitor (PKRi) can restore the deficits in long-term memory and long-term potentiation (LTP) in both mouse models without affecting the Aβ load in the hippocampus. Our results prove the principle that targeting memory enhancing mechanisms can be a valid candidate for developing AD treatment.

**Electronic supplementary material:**

The online version of this article (10.1186/s13041-017-0338-3) contains supplementary material, which is available to authorized users.

## Introduction

Alzheimer’s disease (AD) is a neurodegenerative disorder characterized by cognitive deficits and synaptic dysfunction, for which there is currently no effective treatment available. Genetic studies have shown that mutations in specific set of genes such as *APP*, *PSEN1*, and *PSEN2* are associated with early-onset of familial AD (FAD) [1–3]. *APP* encodes amyloid β (Aβ) precursor protein, while *PSEN1* and *PSEN2* encodes presenilin-1 and presenilin-2, respectively. These proteins are involved in Aβ processing pathway and consequently support a hypothesis that Aβ accumulation in the brain is critical for the onset of AD [4]. In addition to Aβ accumulation, hyper-phosphorylation of tau is another well-known hallmark for AD [5]. Interestingly, both Aβ accumulation and tau hyper-phosphorylation are regulated by eukaryotic translation initiation factor 2α (eIF2α) [6, 7]. Hyper-phosphorylation of eIF2α at Ser 51 is observed in the brains of postmortem AD patients as well as in several AD mouse models [8–11]. In addition, Aβ treatment was shown to induce the phosphorylation of eIF2α in cultured neurons [12]. Whereas the phosphorylation of eIF2α inhibits general mRNA translation, eIF2α phosphorylation enhances translation of the specific group of mRNAs such as β-site APP cleaving enzyme 1 (BACE1) and activating transcriptional factor 4 (ATF4), a suppressor of memory formation by inhibiting cAMP responsive element binding protein (CREB)-dependent transcription [12–14]. Since CREB is essential for long-term memory formation and long-term synaptic plasticity [15–18], reducing eIF2α phosphorylation enhanced long-term potentiation (LTP) and long-term memory by reducing ATF4 translation in mice [19]. In addition to eIF2α, the double-stranded RNA-activated protein kinase (PKR), one of eIF2α kinases, is highly phosphorylated in AD brains [7, 11, 20]. PKR becomes active through the auto-phosphorylation when it binds to ATP and dsRNA [21]. Previous studies revealed that either genetic or pharmacological blockage of PKR enhances LTP and memory in mice [22, 23].

Recent studies have suggested that reducing the phosphorylation level of eIF2α could be one of treatment strategies for AD [9, 10, 13, 24]. Genetic reduction of PERK and GCN2, which are other kinases of eIF2α, ameliorated AD-related phenotypes in synaptic plasticity and behavior in AD mouse models such as APP/PS1 mice and 5XFAD mice [9, 10] (but also see [8]). However, most of the previous studies focused on eIF2α signaling pathway in mainly relation to the production of Aβ [8–10, 13].

We hypothesized that PKR inhibition may enhance synaptic plasticity and subsequently rescue memory deficits in AD mouse models even at late stage of the disease. We used Aβ_1–42_-injected wild-type mice and 5XFAD transgenic mice as acutely induced and genetic model of AD, respectively [25, 26]. Our data showed that PKR inhibitor (PKRi) restored LTP deficit in both AD mouse models. Moreover, we found that PKRi treatment rescued the hippocampus-dependent memory deficits in both mouse models. In addition, acute PKR inhibition did not cause any change in Aβ load in the hippocampus of 5XFAD mice. Taken together, this study suggests that enhancing synaptic plasticity by targeting PKR-eIF2α signaling pathway can be a potential therapeutic target for AD.

## Results

### PKRi treatment rescues the contextual fear memory deficit in 5XFAD mice

**Fig. 1:**
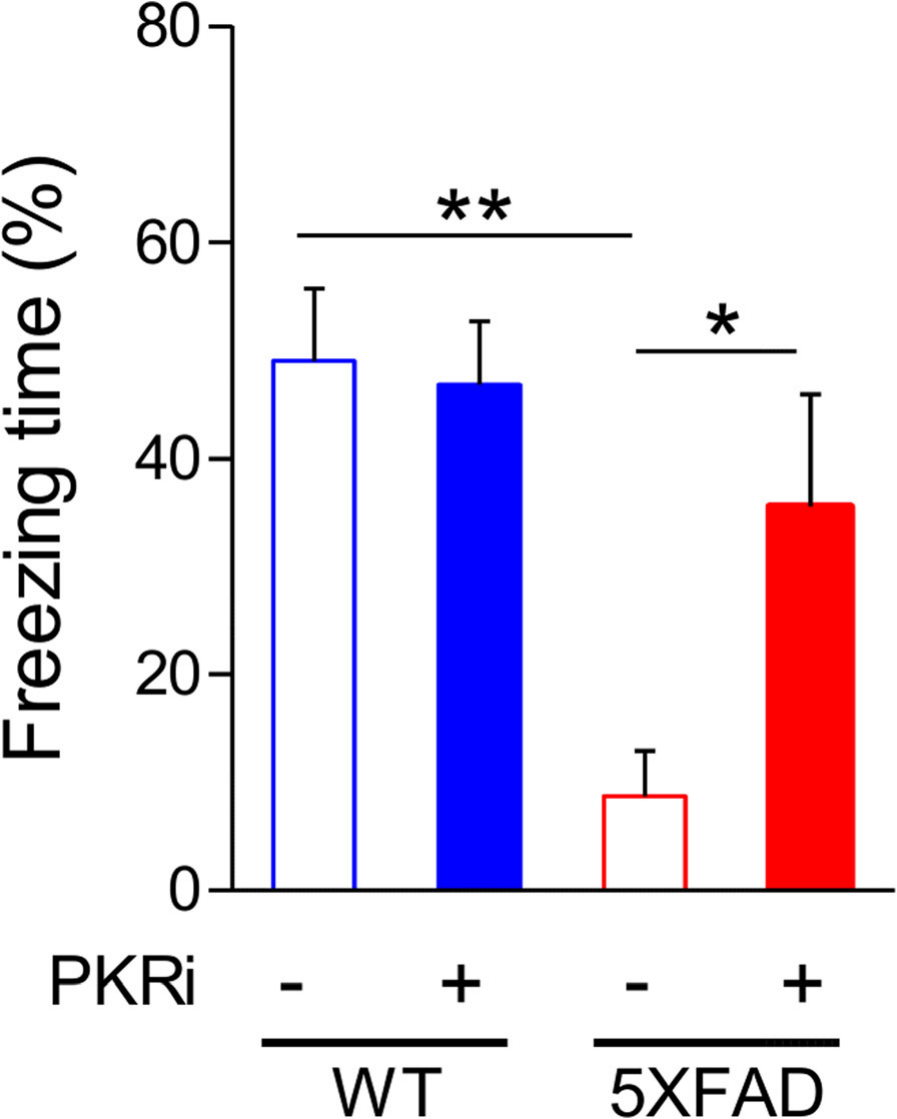
PKRi treatment rescues fear memory deficit in 5XFAD mice. Eleven to twelve months old 5XFAD mice showed significant deficit in contextual fear memory, which was rescued by PKRi treatment (0.335 mg/kg) (% freezing: WT, 49.03 ± 6.67%, *n* = 9 mice; WT + PKRi, 46.78 ± 5.90%, *n* = 10 mice; 5XFAD, 8.74 ± 4.18%, *n* = 6 mice; 5XFAD + PKRi, 35.55 ± 10.38%, *n* = 7 mice; Two-way ANOVA, interaction between genotype and PKRi, *p* = 0.0558, Bonferroni post-tests, *p < 0.05, **p < 0.01). Bars represent as mean ± SEM

We first examined whether PKR inhibitor (PKRi, C-16) treatment can reverse memory deficits in 5XFAD mice which overexpress human mutant forms of APP and PS1 [25]. It is well known that 5XFAD mice show Aβ deposition as early as 2 months after birth and exhibit deficits in memory and LTP after 6 months old [25, 27]. We tested 5XFAD mice (~ 12 months old) in contextual fear conditioning (CFC) which is a hippocampus-dependent associative learning and memory task [28–30]. 5XFAD mice showed profound contextual fear memory deficit at 24 h after training compared with wild-type (WT) littermates (Fig. 1). Interestingly, PKRi treatment (i.p. 0.335 mg/kg, 20 min before training) significantly enhanced freezing in 5XFAD mice without affecting the freezing level in WT littermates (Fig. 1; % freezing: WT, 49.03 ± 6.67%, *n* = 9 mice; WT + PKRi, 46.78 ± 5.90%, *n* = 10 mice; 5XFAD, 8.74 ± 4.18%, *n* = 6 mice; 5XFAD + PKRi, 35.55 ± 10.38%, *n* = 7 mice; Two-way ANOVA, interaction between genotype and PKRi, F(1, 29) =2.19, *p* = 0.056; Bonferroni post-tests, *p < 0.05, **p < 0.01), demonstrating that PKR inhibition can rescue the memory deficit in the transgenic mouse model of AD even when the mice are one year old.

### PKR inhibition restores hippocampal synaptic plasticity in 5XFAD mice

**Fig. 2:**
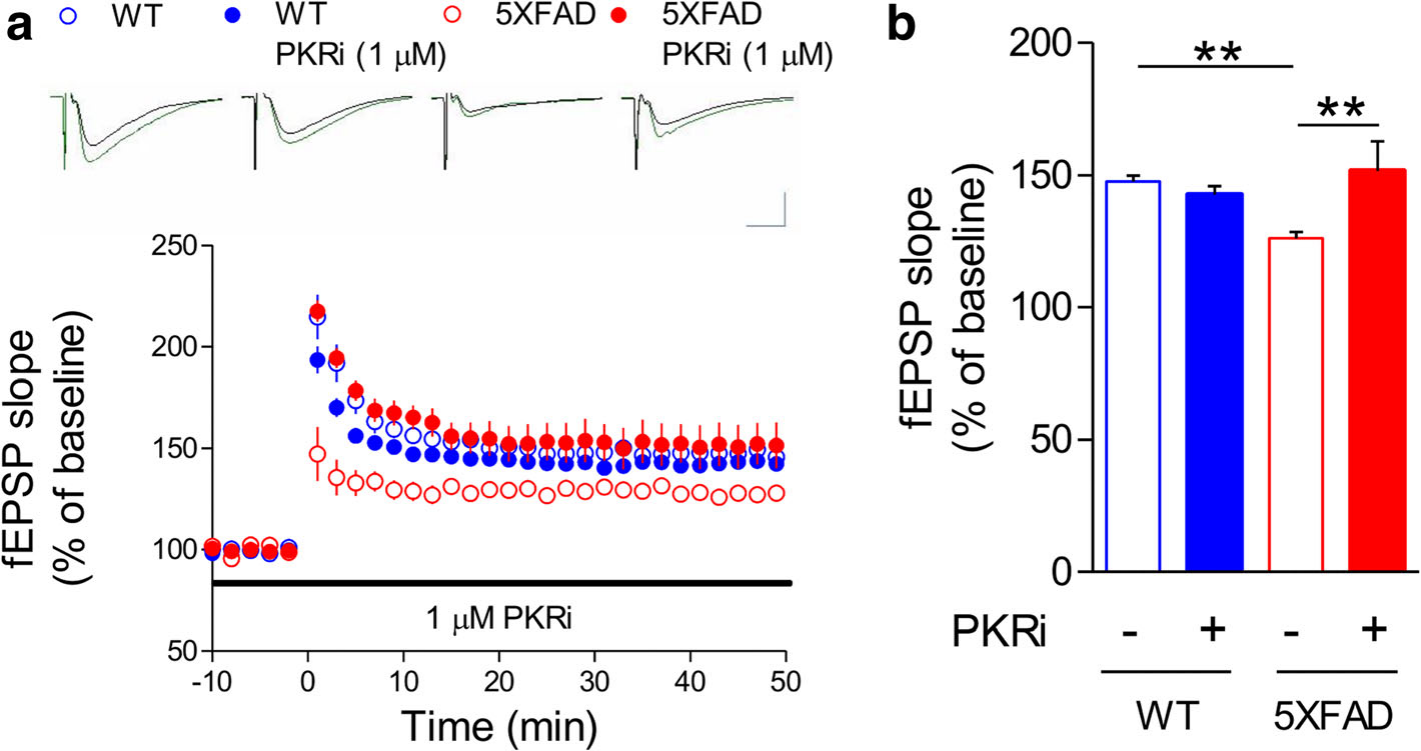
Inhibition of PKR restores LTP impairment in 5XFAD mice. **a** LTP in Schaffer-collateral-CA1 pathway was induced by theta burst stimulation (TBS). Field excitatory synaptic potential (fEPSP) slopes were normalized by the average of baseline recordings. Slices from 5XFAD mice showed significantly reduced LTP than WT, which can be restored by PKRi treatment (1 μM, 90 min). Representative traces were shown above. Black, baseline; Green, average between 40 and 50 min after TBS. Vertical bar, 1.0 mV; horizontal bar, 5 ms. **b** Cumulative data showing the average field excitatory synaptic potential (fEPSP) slope of 40–50 min after TBS (WT, 147.77 ± 2.19%, n = 6 slices from 4 mice; WT + PKRi, 142.83 ± 3.10%, *n* = 9 slices from 5 mice; 5XFAD, 126.22 ± 2.36%, n = 7 slices from 5 mice; 5XFAD and PKRi, 151.67 ± 11.20%, *n* = 5 slices from 3 mice; Two-way ANOVA, interaction between genotype and PKRi, *p < 0.05, Two-way ANOVA, Bonferroni post-tests, **p < 0.01). Bars represent as mean ± SEM

Long-term potentiation (LTP) is considered as a cellular mechanism for long-term memory [31]. Accordingly, LTP deficits have been reported in AD mouse models including 5XFAD mice [9, 27]. We examined whether PKRi can also reverse the LTP deficit in the hippocampal Schaffer-collateral pathway in 5XFAD mice. PKRi (1 μΜ, 0.002% DMSO) was treated from 40 min before LTP induction (theta burst stimulation, TBS: 4 pulses at 100 Hz, 200 ms inter-burst intervals) and throughout the recording. We found that PKRi treatment restored the deficit in TBS-induced LTP in hippocampal slices from 5XFAD mice without affecting LTP in wild-type slices (Fig. 2; Average fEPSP slope, last 10 min: WT, 147.77 ± 2.19%, *n* = 6 slices from 4 mice; WT and PKRi, 142.83 ± 3.10%, *n* = 9 slices from 5 mice; 5XFAD, 126.22 ± 2.36%, *n* = 7 slices from 5 mice; 5XFAD and PKRi, 151.67 ± 11.20%, *n* = 5 slices from 3 mice; Two-way ANOVA, interaction between genotype and PKRi, F(1, 23) = 9.997, *p < 0.05; Bonferroni post-tests; **p < 0.01). In addition to LTP, we also examined whether basal synaptic properties are altered in 5XFAD mice (Additional file 1: Fig. S2). Input-output (I-O) relationship analysis showed that 5XFAD mice have significantly reduced basal synaptic transmission (Additional file 1: Fig. S2A; Two-way ANOVA, Bonferroni post-tests, WT vs 5XFAD, ***p < 0.001 in the range of 40–100 μA; WT, *n* = 25 slices from 11 mice; 5XFAD, *n* = 16 slices from 8 mice), whereas the presynaptic fiber volley amplitudes were not different among groups (Additional file 1: Figure S2B). Moreover, paired pulse facilitation ratio (PPR) was also significantly reduced in 5XFAD mice compared to wild-type littermates (Additional file 1: Figure S2C; Two-way ANOVA, Bonferroni post-tests; WT vs 5XFAD, *p < 0.05 in 25 and 50 μA; WT, *n* = 22 slices from 11 mice; 5XFAD, *n* = 15 slices from 8 mice). Interestingly, PKRi treatment for 30 min rescued the deficits in basal synaptic transmission and PPR in 5XFAD (I-O: Two-way ANOVA, Bonferroni post-tests; 5XFAD vs 5XFAD + PKRi (1 μM), #*p* < 0.05 in the range of 70–100 μA; 5XFAD, *n* = 16 slices from 8 mice; 5XFAD and PKRi, *n* = 7 slices from 4 mice; Additional file 1: Figure S2A; PPR: Two-way ANOVA, Bonferroni post-tests, 5XFAD vs 5XFAD + PKRi (1 μM), ##*p* < 0.01 in 25 μA, #*p* < 0.05 in 50 μA; 5XFAD, n = 15 slices from 8 mice; 5XFAD and PKRi, n = 7 slices from 4 mice; Additional file 1: Figure S2C).

### PKRi treatment does not decrease Aβ_1–42_ in the hippocampus of 5XFAD mice

**Fig. 3:**
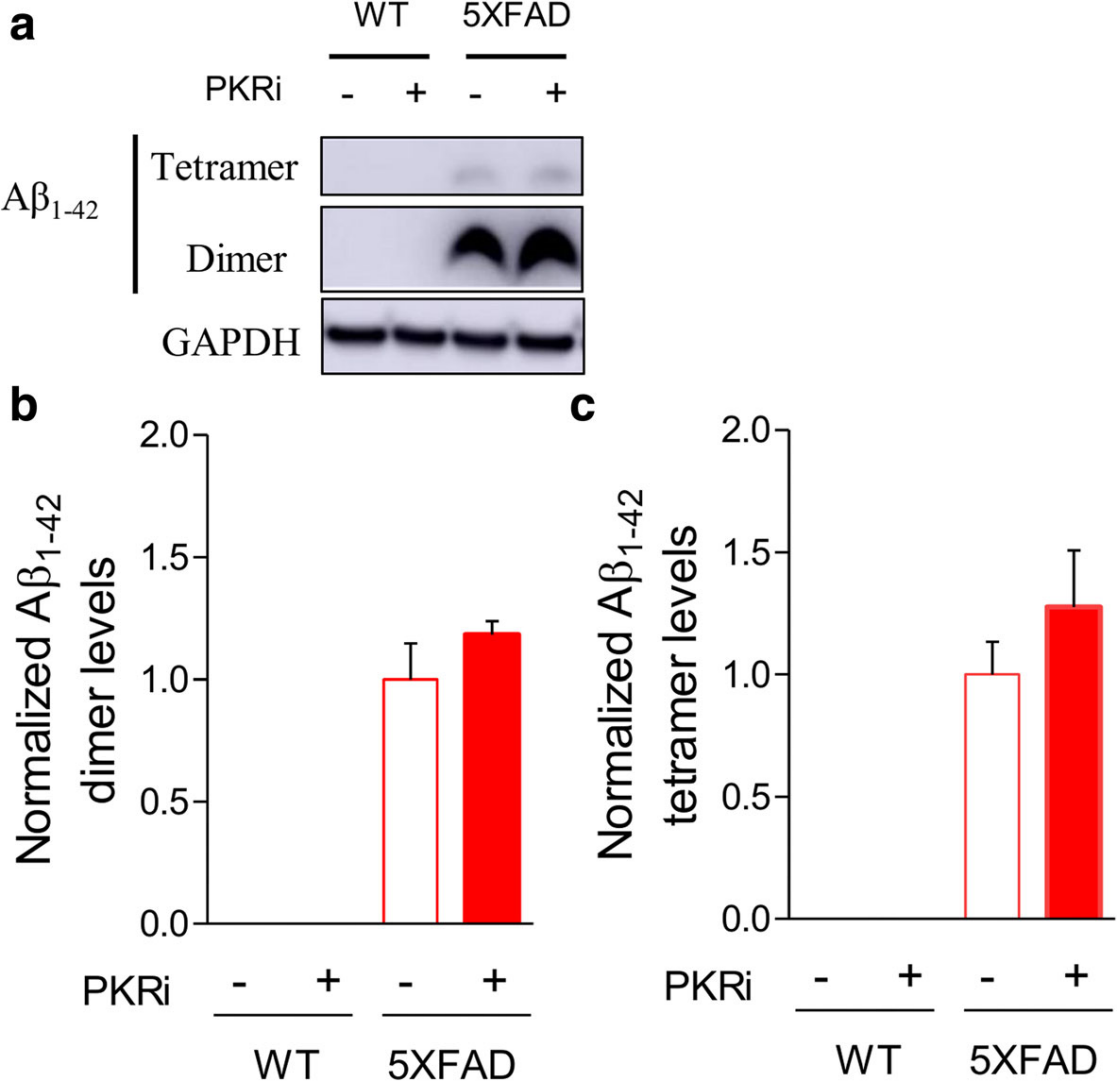
PKRi treatment does not decrease Aβ_1–42_ in the hippocampus of 5XFAD mice. **a** Representative immunoblots of protein extracts from the hippocampi 1 h after PKRi injection (0.335 mg/kg) in WT and 5XFAD mice. **b**, **c** Quantification of the of Aβ_1–42_ oligomers such as dimers and tetramers showing that PKRi treatment did not affect Aβ_1–42_ oligomers in 5XFAD mice (dimer levels normalized by that of 5XFAD; vehicle, 0; vehicle + PKRi, 0; Aβ_1–42_, 1.00 ± 0.15; Aβ_1–42_ + PKRi, 1.18 ± 0.06; unpaired t-test, 5XFAD vs 5XFAD + PKRi, *p* = 0.2674; tetramer levels normalized by that of 5XFAD; vehicle, 0; vehicle + PKRi, 0; Aβ_1–42_, 1.00 ± 0.13; Aβ_1–42_ + PKRi, 1.28 ± 0.23; unpaired t-test, 5XFAD vs 5XFAD + PKRi, *p* = 0.3243; 6 hippocampi from 3 mice per group). Bars represent as mean ± SEM

Previous studies focused on the effect of genetic suppression of eIF2α phosphorylation on Aβ generation [8–10, 13]. We asked whether acute treatment of 5XFAD mice with PKRi reduced Aβ in the hippocampus. We found significantly higher amount of Aβ_1–42_ oligomers such as dimers and tetramers in the hippocampus of 5XFAD mice compared with WT littermates (Fig. 3A). Interestingly, we found that Aβ_1–42_ oligomers were not decreased by PKRi treatment (Fig. 3B, C; dimer levels normalized by that of vehicle-injected 5XFAD; vehicle, 0; vehicle + PKRi, 0; Aβ_1–42_, 1.00 ± 0.15; Aβ_1–42_ + PKRi, 1.18 ± 0.06; unpaired t-test, 5XFAD vs 5XFAD + PKRi, *p* = 0.2674; tetramer levels normalized by that of vehicle-injected 5XFAD; vehicle, 0; vehicle + PKRi, 0; Aβ_1–42_, 1.00 ± 0.13; Aβ_1–42_ + PKRi, 1.28 ± 0.23; unpaired t-test, 5XFAD vs 5XFAD + PKRi, *p* = 0.3243; 6 hippocampi from 3 mice per group). These findings suggest that the acute effect of PKRi on LTP and memory is not based on regulating the amyloidogenesis.

### PKR inhibition rescues memory deficit in Aβ_1–42_-injected mice

**Fig. 4:**
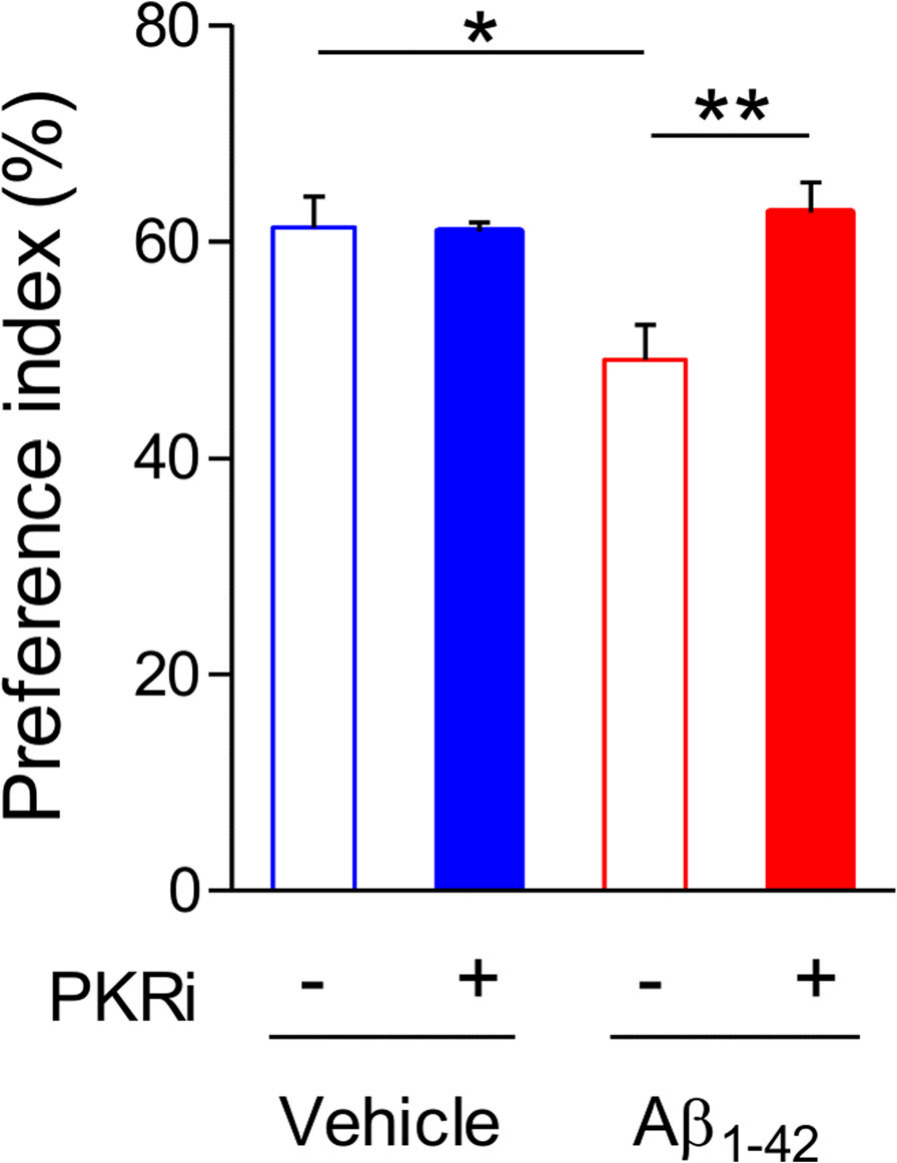
PKRi treatment rescues memory deficit in novel object recognition (NOR) in Aβ_1–42_–injected mice. Injection of Aβ_1–42_ oligomers (3 μg/mouse) induced NOR memory deficit, which was rescued by PKRi treatment. PKRi (0.335 mg/kg) was intraperitoneally injected 20 min before NOR training (Preference index for the novel object: Vehicle, 61.33 ± 2.85%; PKRi, 60.92 ± 0.83%; Aβ, 49.09 ± 3.21%; Aβ_1–42_ and PKRi, 62.7 ± 2.79%; Two-way ANOVA, interaction between Aβ_1–42_ and PKRi, *p < 0.05; Bonferroni post-tests, *p < 0.05, **p < 0.01, n = 6 mice for each group). Bars represent as mean ± SEM

To investigate whether PKR inhibition can be a general strategy to restore synaptic plasticity and memory in multiple AD mouse models, we examined the effect of PKRi on memory in Aβ_1–42_-injected mice. We tested contextual fear memory in vehicle- or Aβ_1–42_-injected wild-type ICR mice (Additional File 1: Figure S3). Unexpectedly, we found that the freezing levels of both groups were low, which makes it difficult to compare freezing levels among different groups (3 μg/3 μl Aβ_1–42_, i.c.v. injection, Vehicle, *n* = 8 mice, 24 h, 6.23 ± 2.21%; Aβ_1–42_, n = 8 mice, 24 h, 7.359 ± 5.79%). Therefore, we used the novel object recognition (NOR) task, which has been frequently used to examine AD-related memory deficits in mice [32]. Since the same mice can be repeatedly tested by replacing object set and long-term memory can be acquired by a single training trial, NOR is frequently used to test effects of pharmacological interventions on learning and memory [33]. We trained the mice in NOR task 2 days after Aβ_1–42_ infusion and tested long-term memory 24 h after the training. As previously reported [34], Aβ_1–42_-injected mice showed significant NOR memory deficit compared to vehicle-injected control mice (Fig. 4). Importantly, PKR inhibitor (PKRi, 0.335 mg/kg) treatment 20 min before the training significantly improved the long-term NOR memory in Aβ_1–42_-injected mice (Fig. 4; preference index for the novel object: Vehicle, 61.33 ± 2.86%; PKRi, 60.92 ± 0.84%; Aβ, 49.09 ± 3.21%; Aβ_1–42_ and PKRi, 62.70 ± 2.80%; Two-way ANOVA, interaction between Aβ_1–42_ and PKRi, F(1, 10) = 9.067, *p < 0.05; Bonferroni post-tests, *p < 0.05, **p < 0.01, *n* = 6 mice for each group), suggesting that inhibiting PKR during training can rescue long-term memory deficit in Aβ_1–42_-injected mice.

### PKR inhibition restores the Aβ_1–42_-induced LTP impairment in hippocampus

**Fig. 5:**
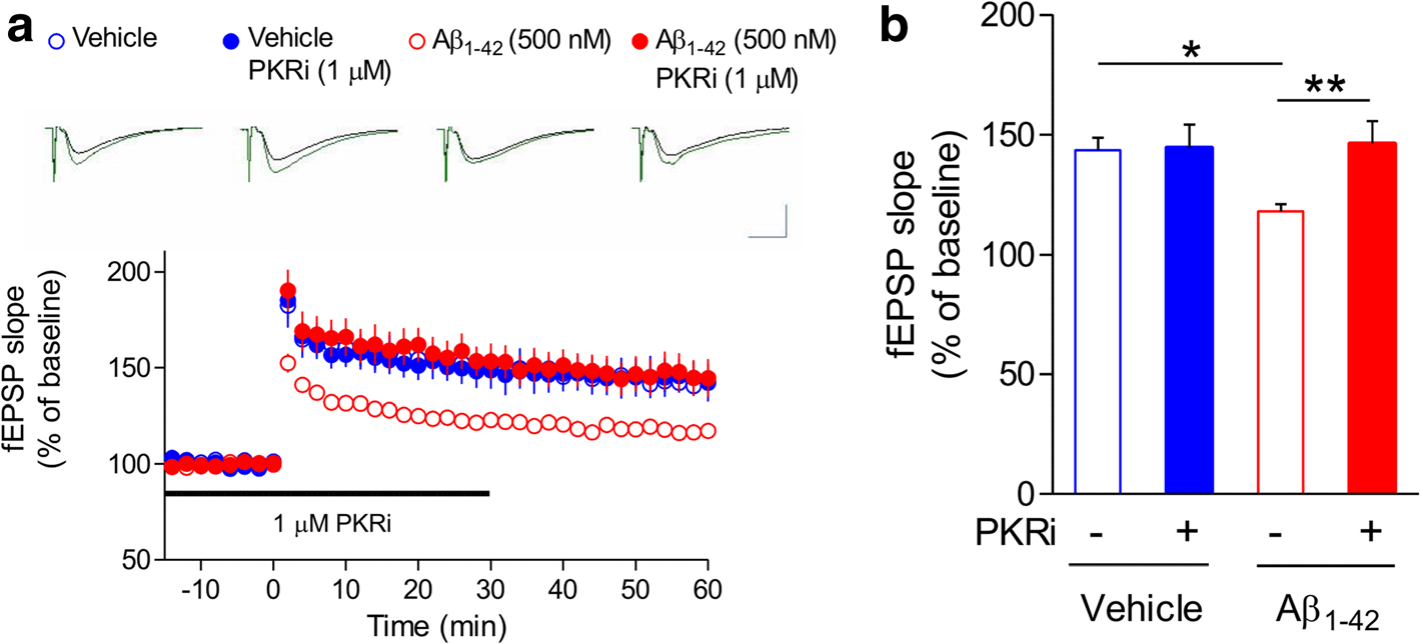
Inhibition of PKR restores Aβ_1–42_–induced LTP impairment in hippocampus**. a** PKRi treatment rescued the LTP deficit in Aβ_1–42_-treated slices. Aβ_1–42_ (500 nM) was treated for 2 h before recording and PKRi (1 μM) was applied for 1 h (30 min before/after LTP induction). Representative traces were shown above. Black, baseline; Green, average between 40 and 50 min after HFS. Vertical bar, 1.0 mV; horizontal bar, 5 ms. **b** Cumulative data showing the average field excitatory synaptic potential (fEPSP) slope of 50–60 min after LTP induction (2X HFS) (Vehicle, 143.52 ± 5.22%, n = 7 slices from 6 mice; PKRi, 144.48 ± 9.73%, n = 7 slices from 5 mice; Aβ_1–42_, 118.00 ± 2.99%, *n* = 12 slices from 8 mice; Aβ_1–42_ and PKRi, 146.28 ± 9.45%, n = 7 slices from 7 mice; Two-way ANOVA, interaction between Aβ_1–42_ and PKRi, *p < 0.05, Two-way ANOVA, Bonferroni post-tests, *p < 0.05, **p < 0.01). Bars represent as mean ± SEM

As previously reported, Aβ_1–42_-treated hippocampal slices showed impaired LTP compared to vehicle–treated slices (Fig. 5A, B) [35, 36]. To test the effect of PKR inhibition on LTP, PKRi (1 μΜ, 0.002% DMSO) was treated from 30 min before LTP induction (2X 100 Hz stimulation, 30 s interval) until 30 min after LTP induction. We found that PKRi treatment significantly enhanced LTP in Aβ_1–42_-treated hippocampal slices whereas it did not affect LTP in control slices (Vehicle, 143.52 ± 5.22%, *n* = 7 slices from 6 mice; PKRi, 144.48 ± 9.73%, n = 7 slices from 5 mice; Aβ_1–42_, 118.00 ± 2.99%, *n* = 12 slices from 8 mice; Aβ_1–42_ and PKRi, 146.28 ± 9.45%, n = 7 slices from 7 mice; Two-way ANOVA, interaction between Aβ_1–42_ and PKRi, F(1, 23) = 4.213, *p < 0.05; Bonferroni post-tests, *p < 0.05, **p < 0.01), suggesting that PKR inhibition can rescue LTP deficit in Aβ_1–42_-treated hippocampus (Fig. 5A, B). Neither Aβ_1–42_ nor PKRi treatment affected basal synaptic properties including I-O relationship, fiber volley amplitude and PPR (Additional file 1: Figure S4).

### PKRi treatment has a trend to reverse Aβ_1–42_-mediated changes in PKR signaling

**Fig. 6:**
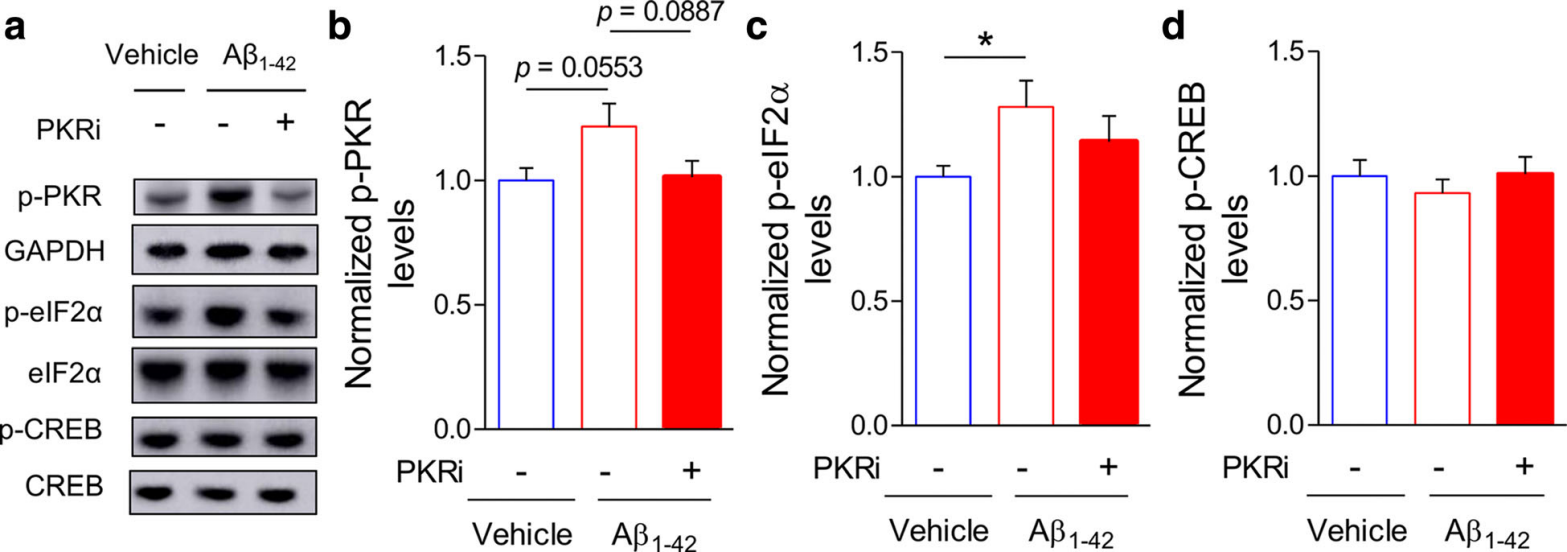
PKRi treatment has a trend to reverse Aβ_1–42_-mediated changes in PKR signaling. **a** Representative immunoblots of protein extracts from hippocampi 30 min after PKRi injection (0.335 mg/kg) in Aβ_1–42_-treated mice. **b** PKRi treatment showed a trend to decrease eIF2α phosphorylation in Aβ_1–42_-treated mice, but the effect was not statistically significant (normalized p-PKR, vehicle, 1.00 ± 0.05, 14, 14 hippocampi from 11 mice; Aβ_1–42_, 1.22 ± 0.09, 15 hippocampi from 13 mice; Aβ_1–42_ + PKRi, 1.01 ± 0.06, 14 hippocampi from 11 mice; unpaired t-test, vehicle vs Aβ_1–42_, *p* = 0.055; Aβ_1–42_ vs Aβ_1–42_ + PKRi, *p* = 0.089). **c** PKRi treatment showed a trend to decrease eIF2α phosphorylation in Aβ_1–42_-treated mice, but the effect was not statistically significant (normalized p-eIF2α, vehicle, 1.00 ± 0.04, 17 hippocampi from 11 mice; Aβ_1–42_, 1.28 ± 0.11, 19 hippocampi from 13 mice; Aβ_1–42_ + PKRi, 1.14 ± 0.10, 18 hippocampi from 12 mice; unpaired t-test, vehicle vs Aβ_1–42_, *p < 0.05; Aβ_1–42_ vs Aβ_1–42_ + PKRi, *p* = 0.354). (D) CREB phosphorylation was slightly reduced by Aβ_1–42_ and was rescued by PKRi treatment although it was not statistically significant (normalized p-CREB, vehicle, 1.00 ± 0.07, 15 hippocampi from 9 mice; Aβ_1–42_, 0.93 ± 0.05, 15 hippocampi from 9 mice; Aβ_1–42_ + PKRi, 1.01 ± 0.07, 16 hippocampi from 10 mice; unpaired t-test, vehicle vs Aβ_1–42_, *p* = 0.426; Aβ_1–42_ vs Aβ_1–42_ + PKRi, *p* = 0.390). Bars represent as mean ± SEM

In order to provide insight into the molecular mechanism underlying PKRi-induced restorations of memory and LTP, we analyzed the phosphorylation levels of PKR, eIF2α and CREB by performing western blot analyses (Fig. 6A). The level of PKR phosphorylation was increased by Aβ_1–42_ and was reversed by PKRi treatment although the effect of PKRi was not statistically significant (Fig. 6B; normalized p-PKR, vehicle, 1.00 ± 0.05, 14 hippocampi from 11 mice; Aβ_1–42_, 1.22 ± 0.09, 15 hippocampi from 13 mice; Aβ_1–42_ + PKRi, 1.01 ± 0.06, 14 hippocampi from 11 mice; unpaired t-test, vehicle vs Aβ_1–42_, *p* = 0.055; Aβ_1–42_ vs Aβ_1–42_ + PKRi, *p* = 0.089). Consistently, eIF2α phosphorylation (p-eIF2α) was significantly increased in Aβ_1–42_-treated mice (Fig. 6C; normalized p-eIF2α, vehicle, 1.00 ± 0.04; Aβ_1–42_, 17 hippocampi from 11 mice, 1.28 ± 0.11, 19 hippocampi from 13 mice; unpaired t-test, *p < 0.05). Importantly, PKRi treatment mildly decreased eIF2α phosphorylation in Aβ_1–42_-treated mice but the effect did not reach the statistical significance (Fig. 6C; Aβ_1–42_, 1.28 ± 0.11, 19 hippocampi from 13 mice; Aβ_1–42_ + PKRi, 1.14 ± 0.10, 18 hippocampi from 12 mice; unpaired t-test, Aβ_1–42_ vs Aβ_1–42_ + PKRi, *p* = 0.354). Neither Aβ_1–42_ nor PKRi treatment did not cause any significant change in CREB phosphorylation (p-CREB) levels, although Aβ_1–42_ treatment slightly decreased p-CREB level (Fig. 6D; normalized p-CREB, vehicle, 1.00 ± 0.07, 15 hippocampi from 9 mice; Aβ_1–42_, 0.93 ± 0.05, 15 hippocampi from 9 mice; Aβ_1–42_ + PKRi, 1.01 ± 0.07, 16 hippocampi from 10 mice; unpaired t-test, vehicle vs Aβ_1–42_, *p* = 0.426; Aβ_1–42_ vs Aβ_1–42_ + PKRi, *p* = 0.390).

## Discussion

AD is a highly heterogeneous disease caused by multiple known and unknown factors. Therefore, it would be extremely difficult to develop treatments by targeting specific causes for individual cases. Based on a hypothesis that manipulating memory-enhancing mechanisms may be beneficial to AD animal models irrespective of their individual etiology [18, 31], we examined whether suppressing PKR/eIF2α signaling can restore synaptic plasticity and behaviors in AD mouse models. It has been previously shown that inhibiting eIF2α phosphorylation can enhance synaptic plasticity and memory in mice [19, 22, 23, 37]. Our results in the electrophysiological recording show that impaired synaptic plasticity can be rescued by PKRi in two different AD mouse models. We assume that these changes in synaptic plasticity consequently contributed to restoring the memory deficit in AD mouse models. Suppressing eIF2α phosphorylation was shown to enhance CREB activity as well as LTP by reducing the translation of ATF4 [19, 38]. It is also known that elevated CREB activity increases the density and complexity of dendritic spines and enhances presynaptic neurotransmitter release [39, 40]. A previous study showed that overexpression of CREB in CA1 rescued spatial memory deficit and altered structure of dendritic spines in 5XFAD mice, which had lower level of CREB phosphorylation [40]. However, we found that p-CREB level was slightly, but not statistically significantly altered by either Aβ_1–42_ or PKRi treatment in the hippocampus under our experimental condition. Although further experiments are required, we speculate that our sample preparation time (2 days after Aβ_1–42_-injection, 30 min after PKRi injection) might not be optimal to observe the impact of Aβ_1–42_-injection and PKR inhibition on CREB phosphorylation.

Previous studies have reported that genetic disruption of PERK in AD mouse models such as APP/PS1 and 5XFAD mice can rescue AD-associated phenotypes, suggesting that inhibiting the upstream kinase of eIF2α may be beneficial to AD [9, 10, 13]. In contrast, there is an inconsistency in the effect of genetic disruption of GCN2 on AD mouse models [8, 9]. Ma and colleagues found that the conditional knockout of GCN2 rescued the deficits in LTP and spatial memory in APP/PS1 mice [9], whereas Devi and Ohno showed that GCN2 deletion could not rescue AD-related phenotypes in 5XFAD mice [8]. Moreover, crossing 5XFAD to *eIF2α* ^S51A^ knock-in line failed to rescue memory deficits in 5XFAD mice [41]. These findings suggest that manipulation of different eIF2α kinases may have distinct impact on cognitive functions in AD mouse models. A recent study showed that PKRi treatment rescued memory deficits in an AD mouse model expressing the human *APOE4* allele, which is consistent with our results [24]. However, to our knowledge, our data is the first showing the beneficial effect of PKRi on synaptic plasticity as well as memory in two independent AD mouse models, which might support the possibility that PKRi could be a potential broad-spectrum drug for AD treatment.

We found that PKR inhibition also reversed the deficits in basal synaptic transmission and short-term plasticity assessed by PPR in 5XFAD mice. Zhu and colleagues recently showed that PKRi treatment in naïve mice decreased GABAergic output of inhibitory networks, resulting the hyperactivity of excitatory neuronal networks [22]. A previous study showed that 5XFAD mice had lower activity of excitatory neural networks compared to their WT littermates [42], which may contribute to the deficits in basal synaptic transmission in 5XFAD mice. We also found that 5XFAD mice showed LTP deficit only when LTP was induced by TBS protocol which is more sensitive to changes in inhibition, but not by high frequency stimulation (100 Hz) protocol (Additional file 1: Figure S5) [43], suggesting an imbalance between excitation and inhibition in 5XFAD mice. It is plausible to speculate that PKRi might have rescued the deficits in basal synaptic transmission and long-term synaptic plasticity by restoring the activity of excitatory networks although it remains to be further investigated. Furthermore, it is worthy to note that changes in inflammation processes involving interferon gamma may underlie the beneficial effect of PKRi on AD mouse models since it has been reported that genetic deletion or inhibition of PKR upregulates the level of interferon gamma, which in turn increases neural excitability and enhances cognitive functions [22, 44].

In contrast to previous studies [19, 22], PKRi treatment did not enhance LTP or learning in our study. Although the reason for the difference is not clear, different experimental conditions such as genetic background of the mice (ICR or B6SJL in our study vs. C57Bl/6J in [19]) may contribute to the difference. Also, Segev and colleagues did not see the memory enhancement in control ApoE3 mice [24].

It is worthy to note that we could rescue the deficits in 12-month-old 5XFAD mice by acute PKRi treatment, suggesting that PKRi might be effective in late stage of AD in spite of the substantial accumulation of amyloid β in the brain. Indeed, we showed that acute PKRi treatment can reverse deficits in LTP and memory in 5XFAD mice without affecting Aβ load in the hippocampus. Considering recent reports on failures in AD clinical trials by targeting amyloid β [45, 46] (but also see [47]), pharmacological interventions enhancing plasticity such as suppressing PKR may provide a promising alternative strategy for developing AD treatment.

## Methods

### Animals

4–6-week-old male ICR mice were purchased from Orient Bio Inc. and B6SJL-Tg (APPSwFlLon, PSEN1*M146 L*L286V)6799Vas/Mmjax mice (5XFAD) were generous gifts from Dr. Woo Keun Song (Gwangju Institute of Science and Technology, Korea) and Dr. Inhee Mook-Jung (Seoul National University College of Medicine, Korea). Both male and female 5XFAD mice were used. Mice were maintained on a 12 h light-dark cycle and food and water were provided ad libitum in vivarium at Seoul National University College of Medicine and Chung-Ang University. Mice were assigned in a group of 4 to 6 per cage and acclimated to the vivarium at least one week before experiments. Prior to experiments, mice were individually handled for 5 min in the testing room each day for 4 days.

### Preparation of Aβ_1–42_ oligomers

Aβ_1–42_ (Abcam, Catalog # ab120301, Lot # APN15158–1-2) peptide was dissolved in 1 ml of hexafluoroisopropanol (HFIP, Sigma) for 24 h on a rocker at room temperature. HFIP was slowly evaporated by using nitrogen gas [36]. Dried Aβ_1–42_ pellet was dissolved in DMSO (final concentration, 4.4 mM, Duchefa, D1370) and immediately frozen with dry ice and stored at −80 °C. In order to oligomerize, Aβ_1–42_ stock was diluted in DPBS (WELGENE, LB001–02) to final DMSO concentrations of 10%, then incubated for 24 h at 37 °C [26]. The Aβ_1–42_ oligomers were analyzed by western blot (Additional file 1: Figure S1) and BCA assay (Thermo pierce).

### PKRi treatment

PKRi (C-16, Cal-biochem, # 527450) stock solution was dissolved in DMSO (670 μg/ml). For behavioral test, PKRi were further diluted in distilled water to a final DMSO concertation of 10% immediate before i.p. injection (0.335 mg/kg body weight). For control group, 10% DMSO in distilled water was used as vehicle. For electrophysiology, PKRi was diluted in ACSF to 1 μM.

### Stereotaxic surgery

Mice were anesthetized with the mixture of ketamine (133 mg/kg) and xylazine (10.5 mg/kg) in saline (i.p. injection). Aβ_1–42_ oligomers (3 μg/3 μl) were injected intracerebroventricularly (I.C.V., AP = −0.1 mm, ML = +1.0 mm from bregma, DV = −2.6 mm from skull) through Hamilton syringe (26 gauge) [48]. The needle was left for an additional 5 min after injection in the place to ensure the diffusion of Aβ_1–42_.

### Novel object recognition (NOR) task

Prior to Aβ_1–42_ injection, mice were habituated to a test arena (33 cm × 33 cm × 33 cm) without an object for 15 min per day for 2 days. Training was performed 2 days after the stereotaxic surgery. During the training session, mice were placed in the test arena containing two identical objects and allowed to explore the objects for 15 min. Twenty-four hours later, mice were placed again in the same test arena but one of the objects was replaced with a novel object. Behavior was recorded by a video camera. The exploration time to each object was scored manually. The test box was cleaned with 70% ethanol between each trial. The experimenter was blinded to the treatments for all the behavioral tests.

### Fear conditioning

Prior to fear conditioning training, mice were acclimated to the testing room for 1 h. Mice were placed in the fear conditioning chamber (Coulbourn Instruments) for 2 min and received two pairs of a tone (2800 Hz, 85 dB, 30 s) and a co-terminating electric foot-shock (0.7 mA, 2 s) with 30 s intervals. One day after the training, mice were placed again in the chamber to test contextual fear memory for 3 min. The freezing behavior was automatically measured by Freeze Frame software (ActiMetrics, IL, USA). Data from one mouse that had freezing rate of deviation more than 2 standard deviations were excluded from the analysis.

### Electrophysiology

Field excitatory postsynaptic potentials (fEPSPs) were performed as previously described [49]. Sagittal hippocampal slices (400 μm thick) were incubated for at least 1 h in artificial cerebrospinal fluid (ACSF: in mM, 120 NaCl, 3.5 KCl, 2.5 CaCl_2_, 1.3 MgSO_4_, 1.25 NaH_2_PO_4_, 10 D-glucose, 20 NaHCO_3_)-filled chamber and additional 2 h in Aβ_1–42_ (500 nM)-treated ACSF-filled chamber before recording. PKRi (1 μM) (Calbiochem, Merck Millipore, Billerica, MA) dissolved in ACSF was perfused from 30 min before LTP induction to 30 min or 50 min after LTP induction. fEPSPs were recorded with a platinum-iridium electrode placed in the CA1 *striatum radiatum*. Bipolar platinum stimulating electrodes were placed in Schaffer-collaterals. LTP was induced by two times high frequency stimulation (2X HFS; 100 pulses at 100 Hz with 30 s inter-train interval) or 3X theta burst stimulation (TBS; 4 pulses at 100 Hz repeated with 200 ms inter-burst intervals) protocol. To determine whether the magnitude of LTP differed significantly among groups, the average fEPSP slopes of 40–50 or 50–60 min after LTP induction from each group were compared. Data were acquired and analyzed by using WinLTP (WinLTP Ltd., ver 2.20b). The experimenter was blinded to the genotypes and treatments.

### Western blotting

Hippocampi were dissected 30 min after i.p. injection of PKRi in Aβ_1–42_-injected mice and 1 h after PKRi injection in 5XFAD mice. Each hippocampus was homogenized in 150 μl lysis buffer containing 10 mM Tris-HCl (pH 6.8) buffer, 1.6% SDS, protease inhibitor and phosphatase inhibitors. 1 μg of the purified Aβ_1–42_ oligomers for I.C.V. injection or 20–30 μg of protein samples from 5XFAD mice and Aβ_1–42_-injected mice were run on a 4–12% bis tris gel and transferred to a PVDF membrane for Aβ_1–42_ or nitrocellulose membrane for other proteins. After blocking in 5% skim milk in 0.1% TBST, membranes were probed with primary antibody (mouse anti-Aβ (4G8), 1:1000, Biolegend, SIG-39220; rabbit anti-p-eIF2α antibody, 1:1000, Cell signaling, 3597S; rabbit anti-p-PKR, 1:1000, ThermoFisher, 44-668G; rabbit anti-eIF2α, 1:1000, Cell signaling, 5324S; rabbit anti-p-CREB,1:1000, Millipore, 06–519; rabbit anti-CREB, 1:1000, Cell signaling, 9197S; mouse anti-GAPDH, 1:10,000, Millipore, MAB374) overnight at 4 °C. After washing 3 times in 0.1% TBST, membranes were probed with horseradish peroxidase-conjugated secondary IgG for 1 h at room temperature. Signals from membranes were detected by using ECL chemiluminescence substrate kit (Thermo Pierce). Proteins were normalized to GAPDH, and phosphorylated proteins were normalized to their respective total proteins.

### Statistics

Effects of PKRi treatment on different groups were analyzed by using two-way ANOVA followed by appropriate post-hoc tests. Some behavioral, electrophysiological and western blotting data were analyzed by using unpaired two-tailed t-test as indicated in the results section. Data are presented as mean ± standard error of the mean (SEM).

## Additional files

Additional file 1: Figure S1. Confirmation of Aβ_1–42_ oligomerization. **Figure S2.** Inhibition of PKR restores basal synaptic dysregulation in 5XFAD mice. **Figure S3.** ICR mice showed the low standard of the freezing behavior in contextual fear conditioning. **Figure S4.** Neither Aβ_1–42_ nor PKRi affected basal synaptic transmission and short-term synaptic plasticity. **Figure S5.** High frequency stimulation (HFS)-induced LTP is normal in 5XFAD mice. (DOCX 709 kb)

## Abbreviations

ACSF: Artificial cerebrospinal fluid
AD: Alzheimer’s disease
ANOVA: Analysis of variance
ATF4: Activating transcriptional factor 4
Aβ: Amyloid β
CFC: Contextual fear conditioning
CREB: cAMP responsive element binding protein
eIF2α: Eukaryotic translation initiation factor 2α
FAD: Familial Alzheimer’s disease
fEPSP: Field excitatory postsynaptic potential
GAPDH: Glyceraldehyde 3-phosphate dehydrogenase
HFS: High frequency stimulation
i.c.v.: Intracerebroventricular
i.p.: Intraperitoneal
LTP: Long-term potentiation
NOR: Novel object recognition
PKR: Protein kinase RNA-activated
PKRi: PKR inhibitor
PPR: Paired pulse facilitation ratio
SEM: Standard error of the mean
TBS: Theta burst stimulation
