# Supplementary material for: Restoring synaptic plasticity and memory in mouse models of Alzheimer’s disease by PKR inhibition
Source: Mol Brain. 2017 Dec 13;10:57. doi: 10.1186/s13041-017-0338-3 (PMC5727890; doi:10.1186/s13041-017-0338-3)
Supplement: Supplementary file 1 — Confirmation of Aβ1–42 oligomerization. Figure S2. Inhibition of PKR restores basal synaptic dysregulation in 5XFAD mice. Figure S3. ICR mice showed the low standard of the freezing behavior in contextual fear conditioning. Figure S4. Neither Aβ1–42 nor PKRi affected basal synaptic transmission and short-term synaptic plasticity. Figure S5. High frequency stimulation (HFS)-induced LTP is normal in 5XFAD mice. (DOCX 709 kb) [file 13041_2017_338_MOESM1_ESM.docx]

**Additional File 1**

**Title: Restoring synaptic plasticity and memory in mouse models of Alzheimer’s disease by PKR inhibition**

Kyoung-Doo Hwang^1,*^, Myeong Seong Bak^2,3*^, Sang Jeong Kim^2,3,4^, Sangmyung Rhee^1,#^, Yong-Seok Lee^2,3,4#^

**
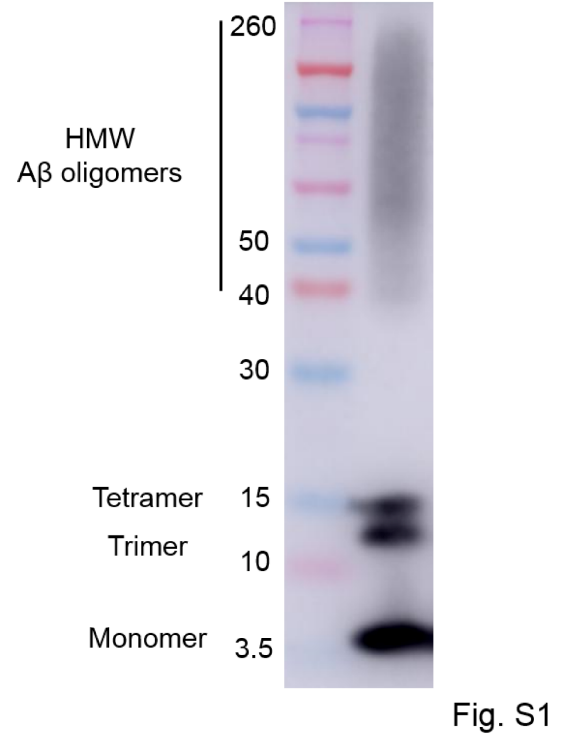
**

**Fig. S1. Confirmation of Aβ_1-42_ oligomerization.** Trimer, tetramer and high molecular weight oligomers of Aβ_1-42_ are detected after oligomerization. Molecular weights are indicated. HMW, high molecular weight.

**
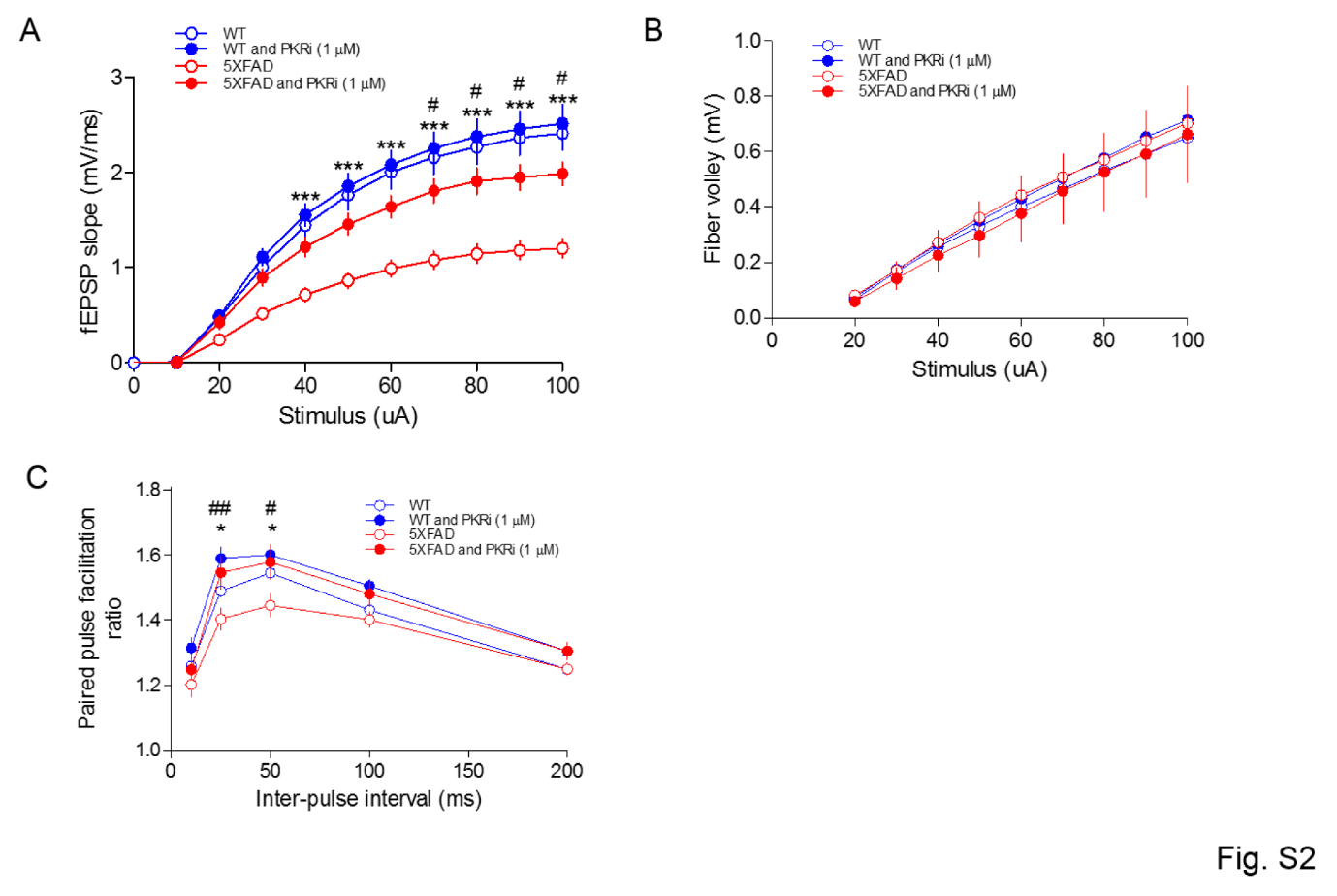
**

**Fig. S2. Inhibition of PKR restores deficits in basal synaptic properties in 5XFAD mice.** (A) Input-output (I-O) curve of fEPSP slopes to stimulus in 5XFAD mouse is impaired compared to their WT littermates but is rescued by PKRi treatment (Two-way ANOVA, Bonferroni post-tests, WT vs 5XFAD, ****p* <0.001 in the range of 40-100 μA, WT vs FAD + PKRi (1 μM), p >0.05, 5XFAD vs 5XFAD + PKRi (1 μM), #*p* <0.05 in the range of 70-100 μA; WT, n = 25 slices from 11 mice; WT + PKRi, n = 17 slices from 10 mice; 5XFAD, n = 16 slices from 8 mice; 5XFAD and PKRi, n = 7 slices from 4 mice). (B) I-O curves of presynaptic fiber volley amplitudes to stimulus have no difference among 4 groups (WT, n = 9 slices from 5 mice; WT + PKRi, n = 14 slices from 9 mice; 5XFAD, n = 9 slices from 7 mice; 5XFAD and PKRi, n = 5 slices from 3 mice; Two-way ANOVA, Bonferroni post-tests, *p* >0.05). (C) Paired pulse facilitation ratio (PPR) was measured by 2 stimuli of different inter-pulse intervals. PPR was impaired in 5XFAD but was rescued by PKRi treatment (Two-way ANOVA, Bonferroni post-tests, WT vs 5XFAD, **p* <0.05 in 25 and 50 μA, WT vs FAD + PKRi (1 μM), *p* >0.05, 5XFAD vs 5XFAD + PKRi (1 μM), ##*p* <0.01 in 25 μA, #*p* <0.05 in 50 μA; WT, n = 22 slices from 11 mice; WT + PKRi, n = 14 slices from 10 mice; 5XFAD, n = 15 slices from 8 mice; 5XFAD and PKRi, n = 7 slices from 4 mice). Bars represent as mean ± SEM.

**
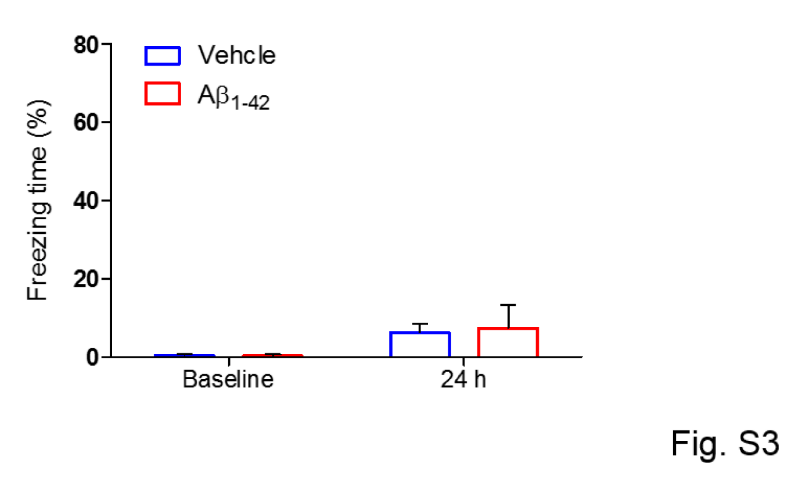
**

**Fig. S3. Low freezing level in ICR mice after fear conditioning.** ICR mice show low levels of freezing level 24 h after training (Vehicle, n = 8 mice, 24 h, 6.23 ± 2.21%; Aβ_1-42_, n = 8 mice, 24 h, 7.359 ± 5.79%). Bars represent as mean ± SEM.

**
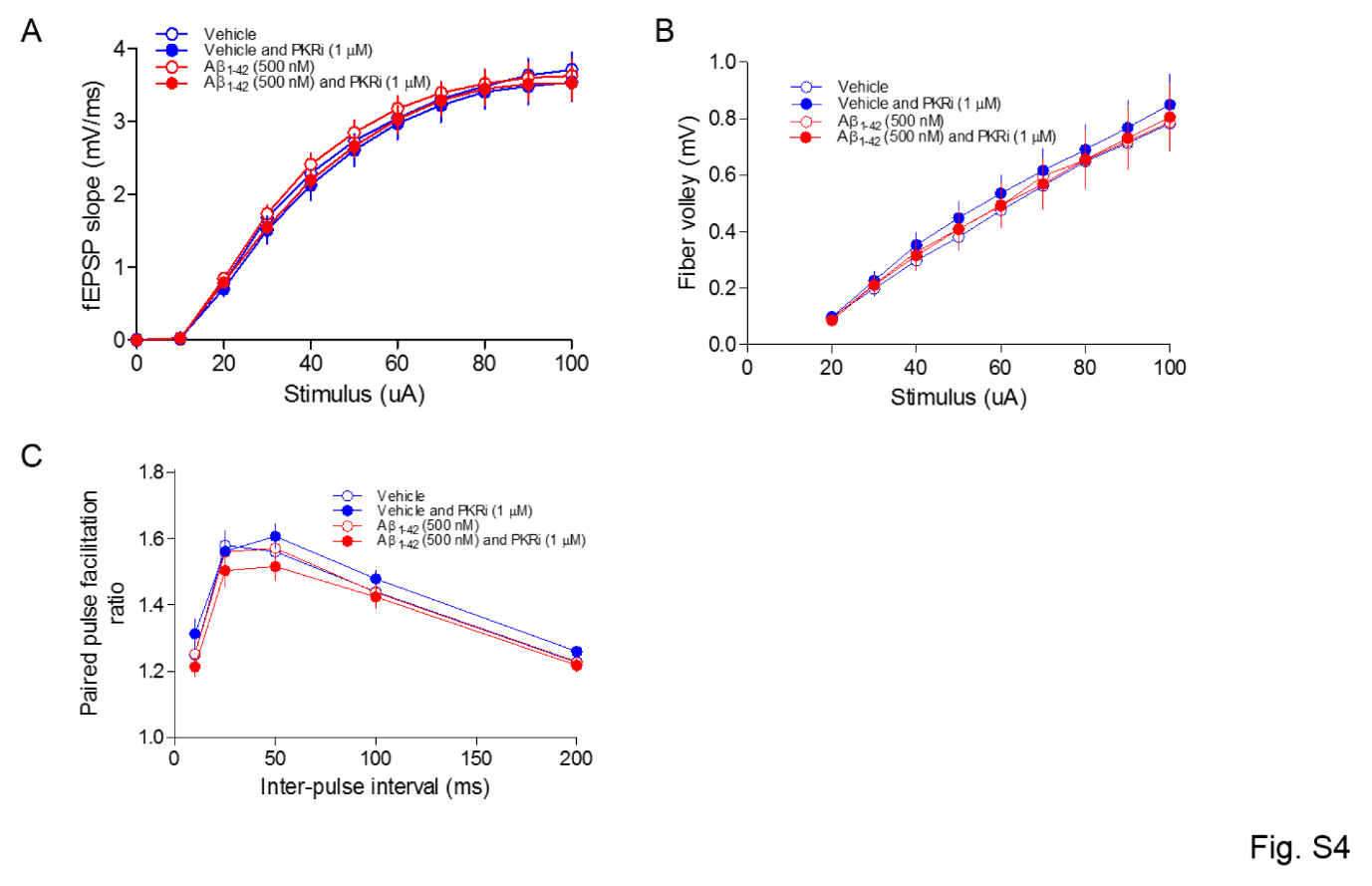
**

**Fig. S4. Neither Aβ_1-42_ nor PKRi affected basal synaptic transmission and short-term synaptic plasticity.** (A) Aβ_1-42_ does not affect Input-output (I-O) curves of fEPSP slopes to stimulus (*p* > 0.05). (B) I-O curves of presynaptic fiber volley amplitudes to stimulus have no difference (*p* > 0.05). (C) Paired pulse facilitation ratio (PPR) measured by 2 stimuli of different inter-pulse intervals shows no difference among groups (Two-way ANOVA, Bonferroni post-tests, *p* >0.05 in all panels; Vehicle, 8 slices from 4 mice; Aβ_1-42_, 8 slices from 4 mice; Vehicle and PKRi, 6 slices from 3 mice; Aβ_1-42_ and PKRi, 6 slices from 3 mice). Bars represent as mean ± SEM.


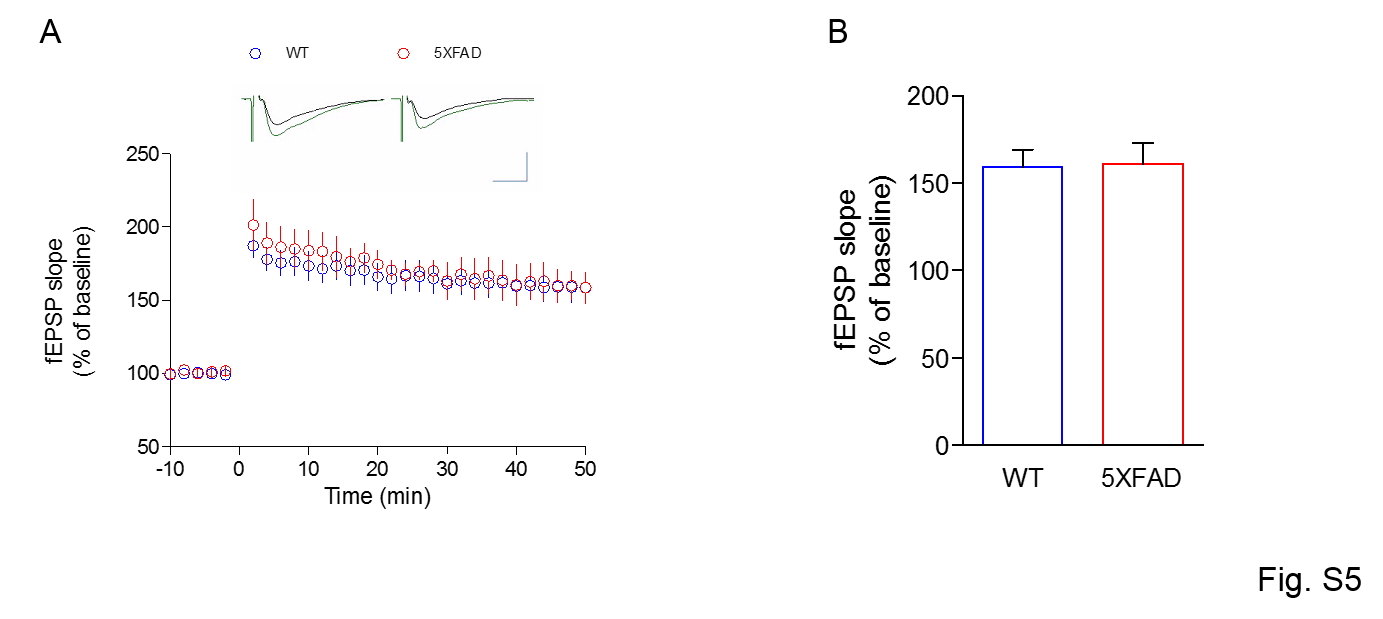


**Fig. S5. High frequency stimulation (HFS)-induced LTP is normal in 5XFAD mice.** (A) LTP in hippocampal CA1 of 5XFAD mice does not show impairment compared to that of their WT littermates. Vertical bar, 1.0 mV; Horizontal bar, 5 ms. (B) Cumulative data showing the average field excitatory synaptic potential (fEPSP) slope of 40-50 min after 2X HFS (WT, 159.10 ± 10.06%, n = 10 slices from 4 mice; 5XFAD, 161.00 ± 11.89%, n = 4 slices from 3 mice; unpaired t-test, *p* = 0.92, N.S., not significant). Bars represent as mean ± SEM.
